# Multiple Linear Dichroism Inversions in SnO Monolayers for Polarization-Sensitive UV Photodetection: An Ab Initio Investigation

**DOI:** 10.1021/acsanm.4c06552

**Published:** 2025-01-30

**Authors:** Michele Re Fiorentin, Francesca Risplendi, Maurizia Palummo, Giancarlo Cicero

**Affiliations:** † Department of Applied Science and Technology, Politecnico di Torino, corso Duca degli Abruzzi 24, 10129 Torino, Italy; ‡ Dipartimento di Fisica and INFN, Università di Roma “Tor Vergata”, via della Ricerca Scientifica 1, 00133 Roma, Italy

**Keywords:** 2D materials, low-symmetry materials, linear dichroism inversion, optoelectronics, density functional theory, BSE-GW calculations, tin monoxide

## Abstract

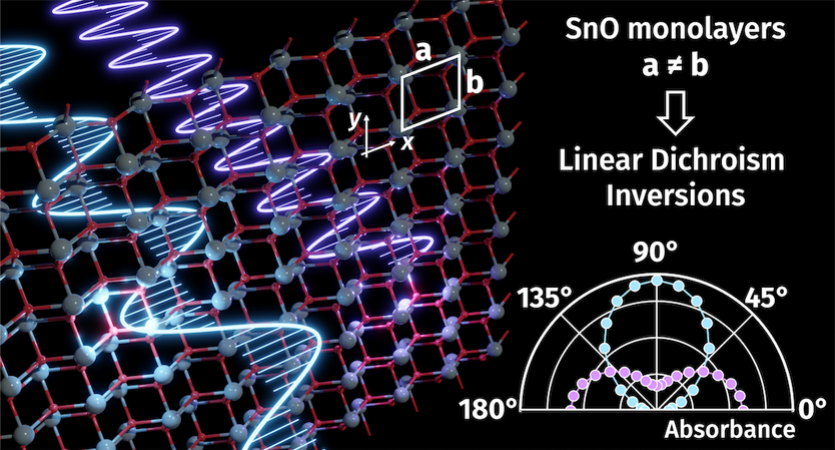

Tin monoxide (SnO) undergoes a phase transition from litharge-like tetragonal (space group *P*4/*nmm*) to orthorhombic geometry (layer group *pmmn*) in passing from multilayer to monolayer crystals. By means of ab initio ground and excited-state methods, we explore the impact of the reduced *pmmn* spatial symmetry on the electronic and optical properties of SnO monolayers. As a consequence of the in-plane anisotropy, the electronic states of the band edges show asymmetric projections onto the p_*x*_ and p_*y*_ atomic orbitals along orthogonal directions in the Brillouin zone. This results in optical absorption and exciton properties that are highly sensitive to the direction of in-plane polarized light. In contrast to typical linear dichroic materials, which generally favor the absorption of one polarization over the orthogonal one across a wide frequency range, we show that SnO monolayers display linear dichroism inversion. Here, the energy ordering of the exciton states causes the two orthogonal polarizations to be absorbed with different intensities depending on the light frequency. We observe multiple inversions of the linear dichroism across wavelengths from 200 to 400 nm. These properties make SnO monolayers promising candidates for further exploration of low-symmetry, two-dimensional materials for advanced applications in polarization-sensitive nanoscale devices. In addition, we propose utilizing optical dichroism measurements as a means to probe the recently predicted ferroelastic-to-paraelastic transition of SnO monolayers.

## 1 Introduction

**Figure 1:**
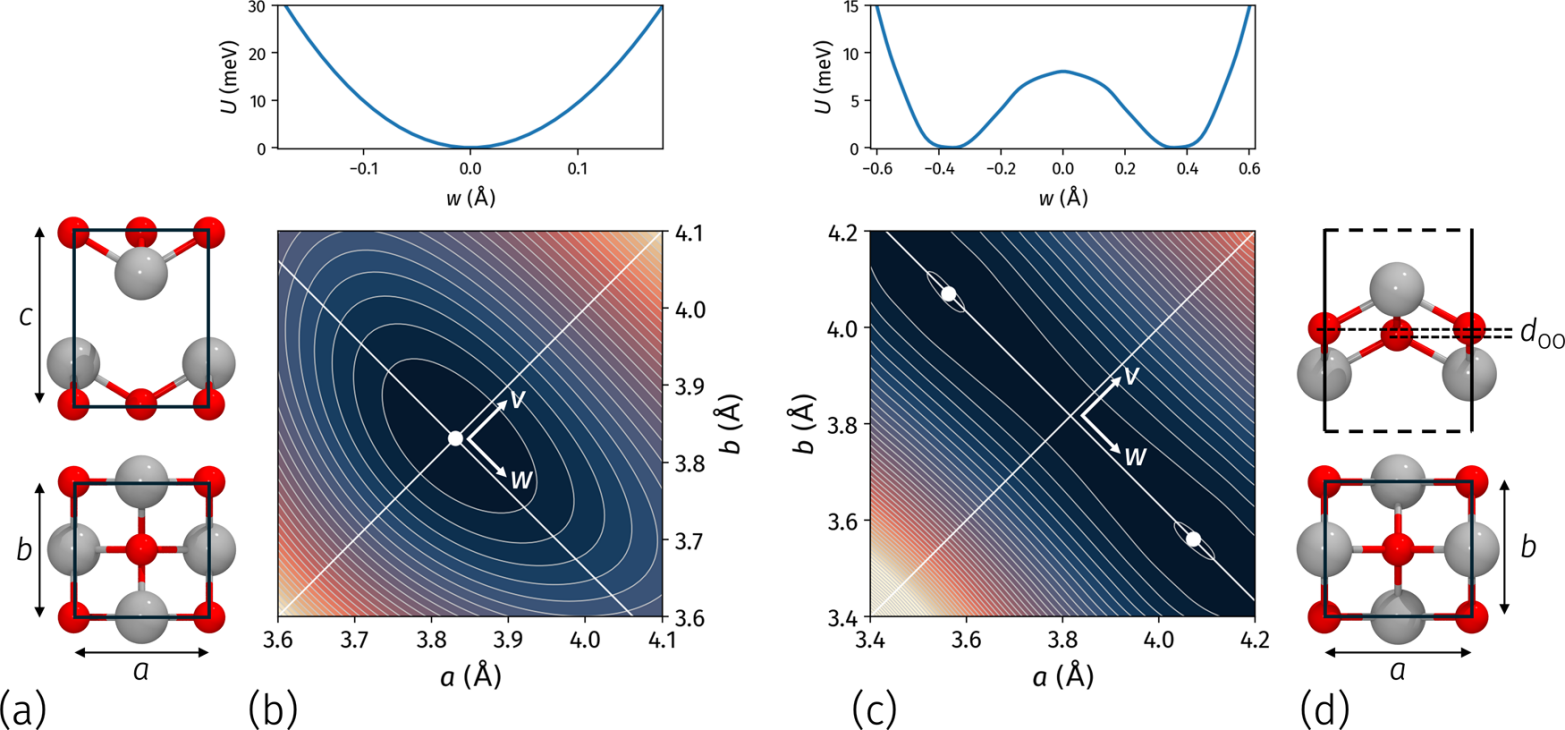
Optimized geometries and total energy contours at varying lattice parameters *a* and *b* for SnO bulk (a, b) and ML (c, d). The upper panels in panels (b) and (c) show cuts of the energy surfaces along direction **w** in the **ab** plane. The vertical distance between oxygen atoms in the ML unit cell, *d*_OO_, is marked in panel (d).

Tin monoxide (SnO) has attracted attention as one of the few intrinsically *p*-doped oxides, showing a carrier mobility comparable to that of *n*-type semiconductors.^1−5^ Bulk SnO adopts a lithargic structure with a tetragonal unit cell. The crystal exhibits 4-fold rotational symmetry about the vertical, **c** axis, as well as mirror symmetries across two planes parallel to **c** and orthogonal to the **ab**-plane; see Figure 1a. Additionally, SnO features a glide plane, being symmetric for reflections through the **ab**-plane, combined with translation by **a**/2 and **b**/2. As a result, the space group is *P*4/*nmm*, nonsymmorphic, with corresponding point group *D*_4*h*_. The layered structure of SnO allows for its downsizing to a few layers, with a concurrent electronic band gap increase from around 0.6 eV, in the bulk, to about 4 eV in few-layer systems.^6^ The tunable band gap, coupled with high hole mobility, makes SnO one of the most interesting transparent oxides^7^ for applications in thin-film transistors,^8−10^ resistive switching memories,^11,12^ photocatalysis,^13,14^ and energy storage.^15,16^

The lithargic tetragonal structure of the bulk is retained when SnO is thinned down to a few layers. However, it has been pointed out that in the monolayer (ML) limit, SnO adopts an othorhombic geometry with layer group *pmmn* and corresponding point group *D*_2*h*_.^17^ This orthorhombic phase has been predicted to be stable at low temperatures below a critical temperature *T*_c_, above which the tetragonal symmetry is restored.^18^ The ferroelastic phase transition is governed by the energy barrier *J* separating the orthorhombic and the tetragonal phases, with *T*_c_ estimated to be approximately 8 K. Although this suggests that the *pmmn* phase is accessible only at very low temperatures, the thermal stability of the ferroelastic phase can be significantly improved through strategies such as doping,^18^ the exploitation of substrate effects,^19^ and strain engineering.^20^ These approaches may facilitate the investigation of the properties of ferroelastic SnO MLs while creating opportunities for their applications.

In its ferroelastic phase, SnO MLs exhibit a 2-fold rotational symmetry around the vertical axis orthogonal to the **ab**-layer plane, along with two vertical mirror planes orthogonal to **ab** and a glide plane, combining a reflection through the layer plane with a translation by **a**/2 and **b**/2, see Figure 1d. As in the bulk case, the presence of the glide plane makes the space group nonsymmorphic. The adoption of an orthorhombic cell and the resulting change from 4-fold (*D*_4*h*_) to 2-fold (*D*_2*h*_) rotational symmetry indicates a significant reduction in symmetry compared to the bulk or even the two-layer counterparts, with intriguing implications for the optoelectronic properties. Indeed, the orthorhombic unit cell and the associated low symmetry of the SnO ML suggest potential anisotropy in the optical absorption of in-plane polarized light. Such a dichroic crystal, with large optical band gap, would then be interesting for UV photodetection, in particular in UV communications.^21−23^ Polarization-sensitive photodetectors in the UV range are relatively uncommon, due to the difficulty in finding materials that exhibit both UV absorption and optical dichroism, especially in the 2D limit.^24^ SnO ML could then represent an effective addition to the group of large-bandgap, low-symmetry 2D semiconductors.^25^

In this article, we study the changes in the electronic band structure and optical absorption of SnO in passing from the bulk phase to the ferroelastic phase of the ML by means of ab initio simulations based on Density Functional Theory (DFT) and many-body perturbation theory (MBPT). Crucially, our investigations reveal that the *pmmn* phase of SnO MLs presents multiple linear dichroism inversion (LDI) for wavelengths between 200 and 400 nm. In conventional dichroic materials, such as black phosphorus,^26,27^ the absorption of light polarized along a specific direction is consistently larger than the absorption of the orthogonal polarization across a wide range of frequencies.^28−31^ Conversely, materials showing LDI will preferentially absorb light with different polarization depending on the frequency. In such cases, it is possible to identify frequency windows where one polarization is absorbed more intensely than the orthogonal one and other ranges where the absorption strength is inverted. LDI has been identified in only a limited number of materials characterized by low symmetry, in particular quasi-1D systems^32^ or 2D materials.^33^ Typically, LDI materials exhibit a single inversion point^34−36^ with only a few samples reported to feature more.^37^ In this study, we demonstrate that SnO MLs display at least six such inversions in the UV range.

The 2D thickness, UV absorption and multiple LDI of SnO ML, combined with its relatively low cost compared to established LDI crystals like PdSe_2_^38^ or PdPS,^37^ make it particularly interesting for the development of optoelectronic devices sensitive to light polarization, such as integrated nanodevices, optical switches, and photodetectors^33,39^ that are able to distinguish more than two frequency ranges in the UV spectrum. Finally, besides the possible applications, the predicted optical properties offer a non-invasive experimental tool for probing the ferroelastic behavior of freestanding SnO MLs.

## 2 Methods

### 2.1 DFT Calculations

DFT calculations were performed with the Quantum ESPRESSO code.^40−42^ We employed the Perdew–Burke–Ernzerhof functional,^43^ and introduced van der Waals interactions through the DFT-D3 approach.^44^ Norm-conserving Troullier-Martins pesudopotentials^45^ and a plane-wave cutoff energy of 80 Ry were used for both systems. A 6 × 6 × 6 (6 × 6 × 1) Monkhorst–Pack *k*-point mesh^46^ was used in the modeling of the bulk (ML) structure. Details regarding the convergence of DFT calculations can be found in the Supporting Information. A 15 Å vacuum region along the direction perpendicular to the layer planes was introduced to ensure the decoupling of the periodic replicas in the ML system. Structure relaxation was assumed at convergence when the maximum component of the residual forces on the ions was smaller than 1 × 10^–4^ Ry/Bohr. As previously reported,^7^ the impact of spin–orbit coupling on the electronic structures is negligible in all systems. The phonon dispersions of SnO ML and bulk are reported in the Supporting Information, Figure S4.

### 2.2 MBPT Calculations

The optoelectronic properties of the optimized structures were studied with the YAMBO code.^47,48^ Quasiparticle (QP) band structures were obtained within the G_0_W_0_ approximation. The Godby–Needs plasmon-pole model^49^ was used. We chose the electronic band gap at the Γ point as a parameter to converge within 50 meV. For the bulk structure, we selected a 6 × 6 × 6 *k*-point mesh. In modeling the ML system, we exploited a recently implemented algorithm of stochastic integration and interpolation of the truncated Coulomb screening in the Brillouin zone (BZ) of 2D materials.^50^ This approach drastically improves convergence with respect to the *k*-point mesh density of 2D systems, and an 8 × 8 × 1 grid was chosen for the ML. 500 bands and Bruneval–Gonze terminators^51^ were used in the calculation of the correlation self-energy. The convergence of the dielectric screening with the number of empty bands, *N*_b_, and the energy cutoff, *E*_G_, on the dielectric matrix was obtained with *N*_b_ = 1000, *E*_G_ = 25 Ry for SnO bulk, and *N*_b_ = 2000, *E*_G_ = 25 Ry in the ML case.

The QP band structures were used to build and diagonalize the Bethe–Salpeter Hamiltonian. For the bulk system, the Bethe–Salpeter equation (BSE) was solved considering an 18 × 18 × 18 *k*-point grid, 50 bands, and a 2 Ry cutoff for the static screening, and 4 valence +4 conduction bands in the BSE kernel. For the ML, a 30 × 30 × 1 *k*-point grid, 100 bands, and a 6 Ry cutoff on the static screening were employed, with 4 valence +5 conduction bands in the BSE kernel.

Further details on the convergence of the MBPT calculations are provided in the Supporting Information.

## 3 Results and Discussion

The geometry optimization of bulk SnO results in the tetragonal structure shown in Figure 1a. By independently varying the in-plane lattice parameters *a* and *b*, we identify a single minimum in the total energy surface at *a* = *b* = 3.831 Å, Figure 1b. The optimized interlayer distance is *c* = 4.785 Å, in line with experimental values.^52,53^ In the litharge structure of SnO, tin atoms are coordinated to four O atoms and lie out of the oxygen plane due to the interaction between the Sn(5s)–O(2p_*z*_) orbitals and the Sn(5p_*z*_) orbitals.^54−56^ This interaction is permitted by the tetragonal symmetry and results in an electron density that projects out of the Sn plane, toward the neighboring layer. Additionally, the Sn(5s + 5p_*z*_) orbitals in one layer hybridize with the corresponding orbitals of the nearest tin atom in the adjacent layer, leading to an interlayer Sn–Sn interaction that affects both the structural and the electronic properties of the material.^57,58^ Indeed, each Sn atom interacts equally with four other Sn atoms in the adjacent layer, imposing a tetragonal geometry. This interlayer Sn–Sn interaction ensures that the *D*_4*h*_ symmetry with *a* = *b* is preserved when SnO is exfoliated to a few layers, down to two. However, in the ML system, the lack of interlayer interaction causes a distortion of the in-plane geometry.^17^ Each Sn–O pair is now free to increase its Sn(5s + 5p)–O(2p) overlap, resulting in the oxygen atom moving closer to Sn both out of and within the plane. This leads to a shorter in-plane distance between atoms along one direction [e.g., **b** in Figure 1d] compared to the other [e.g., **a** in Figure 1d], together with a displacement of the O atoms out of their common plane. Indeed, the total energy surface of SnO ML at varying *a* and *b*, Figure 1c, shows two equivalent minima with *a* = 4.079 Å and *b* = 3.549 Å or vice versa, aligning with calculations.^17^ The comparison of the electronic projected density of states (pDOS) for SnO ML in the ferroelastic phase and in the paraelastic, tetragonal cell with lattice parameter *a* = *b* = 3.82 Å, Supporting Information Figure S2, reveals a stabilization of the Sn–O bond along the *x*-direction in the asymmetric cell, contributing to the overall stabilization of the *pmmn* geometry.

The total energy cut along direction **w**, in the upper panel Figure 1c, shows that the two degenerate minima are separated by an energy barrier *J* ≃ 8 meV, in agreement with the values reported in ref (17) for PBE calculations with van der Waals dispersion. We notice that these values, and the corresponding critical temperature, are larger than what was found in an earlier study.^18^ Indeed, the predicted value of *J*, and, consequently, of the critical temperature, shows strong dependence on the choice of functional, pseudopotential, and treatment of the dispersion forces. The vertical distance between oxygen pairs is *d*_OO_ = 0.28 Å. Consequently, unlike the bulk system, the SnO ML lacks all symmetry operations involving 90° rotations around the vertical axis, and its point group becomes *D*_2*h*_. The asymmetry between the **a** and **b** directions influences the material’s optoelectronic properties, which we explore in the following paragraphs.

**Figure 2:**
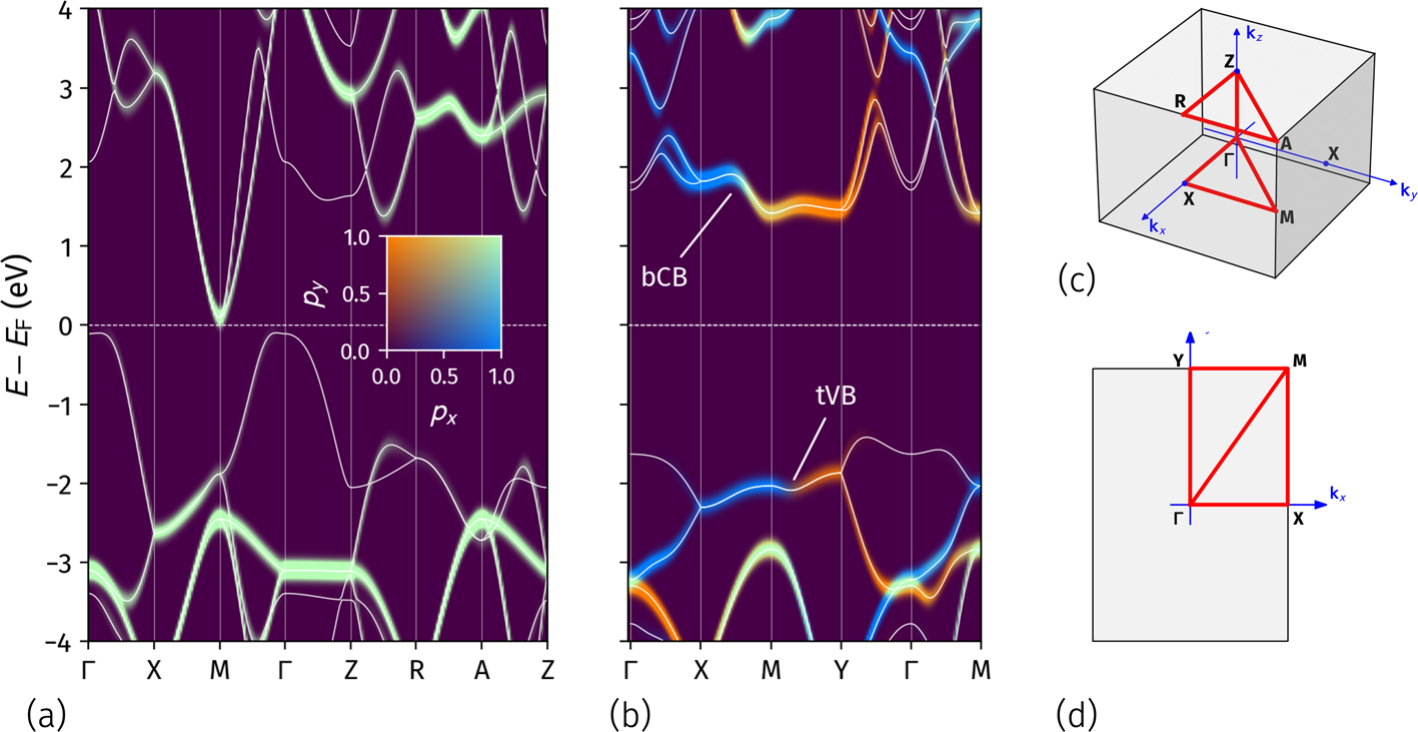
DFT electronic bands of SnO bulk (a) and ML (b) along the high-symmetry paths in the respective BZs reported in panels (c) and (d). Energies are referenced to Fermi energy *E*_F_. Overlaid on the band structures in panels (a) and (b) is the *k*-resolved density of states projected onto p_*x*_ and p_*y*_ orbitals of both Sn and O. The normalized weights of the p_*x*_ and p_*y*_ contributions to each electronic state are highlighted in color shades, as indicated by the color code in the inset of panel (a). The two top valence bands and the two bottom conduction bands are marked by tVB and bCB, respectively, in panel (b).

The different in-plane Sn–O interactions between bulk and ML SnO, which ultimately cause symmetry reduction from *D*_4*h*_ to *D*_2*h*_, are evident in the orbital composition of the electronic band states. In Figure 2a, we show the band structure of SnO bulk, computed at the DFT level, along the high-symmetry path in the BZ shown in panel (c). We align the crystal unit cell to the *x*, *y*, *z* directions in space and, on top of the band structure, we overlay the weights of the projections of the electronic states onto the p_*x*_ and p_*y*_ orbitals of both Sn and O atoms. The orbital contributions are represented by color shades, with blue indicating p_*x*_ and orange representing p_*y*_, as shown in the inset of panel (a). We observe that when present, the bands of SnO bulk consistently show equivalent contributions from both p_*x*_ and p_*y*_, indicated by the light-green shade. This highlights the symmetry between the **a** and **b** directions in real space and, consequently, complete in-plane isotropy.

In the ML, the reduced symmetry results in the rectangular shape of the 2D BZ (Figure 2d). Unlike the bulk case, the midpoint along **k**_*x*_ is not equivalent to its counterpart along **k**_*y*_, and the single X point in the bulk BZ splits into the two inequivalent points X and Y in the ML BZ, c.f. Figure 2c,d. The DFT band structure and *k*-resolved projected density of states (*k*-pDOS) onto p_*x*_ and p_*y*_ orbitals in Figure 2b reveal a clear asymmetry in orbital contributions across the BZ. The two top valence bands (tVB), degenerate along the X̅M̅–M̅Y̅ directions, are mainly contributed by p_*x*_ (blue shade) along the X̅M̅ path, including both symmetry points. Beyond the M point, toward Y, the dominant contribution becomes p_*y*_ (orange shade). The bottom conduction bands (bCB), also degenerate along X̅M̅–M̅Y̅, show a similar behavior, although at and around the M point, both p_*x*_ and p_*y*_ contribute to the electronic states, with a slight preference for p_*y*_. As a consequence of the reduced *D*_2*h*_ symmetry, then, a significant anisotropy in the electronic properties along the **k**_*x*_ and **k**_*y*_ directions in the BZ emerges. Importantly for subsequent analyses, the states of the tVB and bCB at the X and Y points predominantly show p_*x*_ and p_*y*_ character, respectively. At the M point, tVB states are mainly contributed by p_*x*_ orbitals, while the bCB shows mixed character. The full *k*-pDOS of both bulk and ML are reported in the Supporting Information.

**Figure 3:**
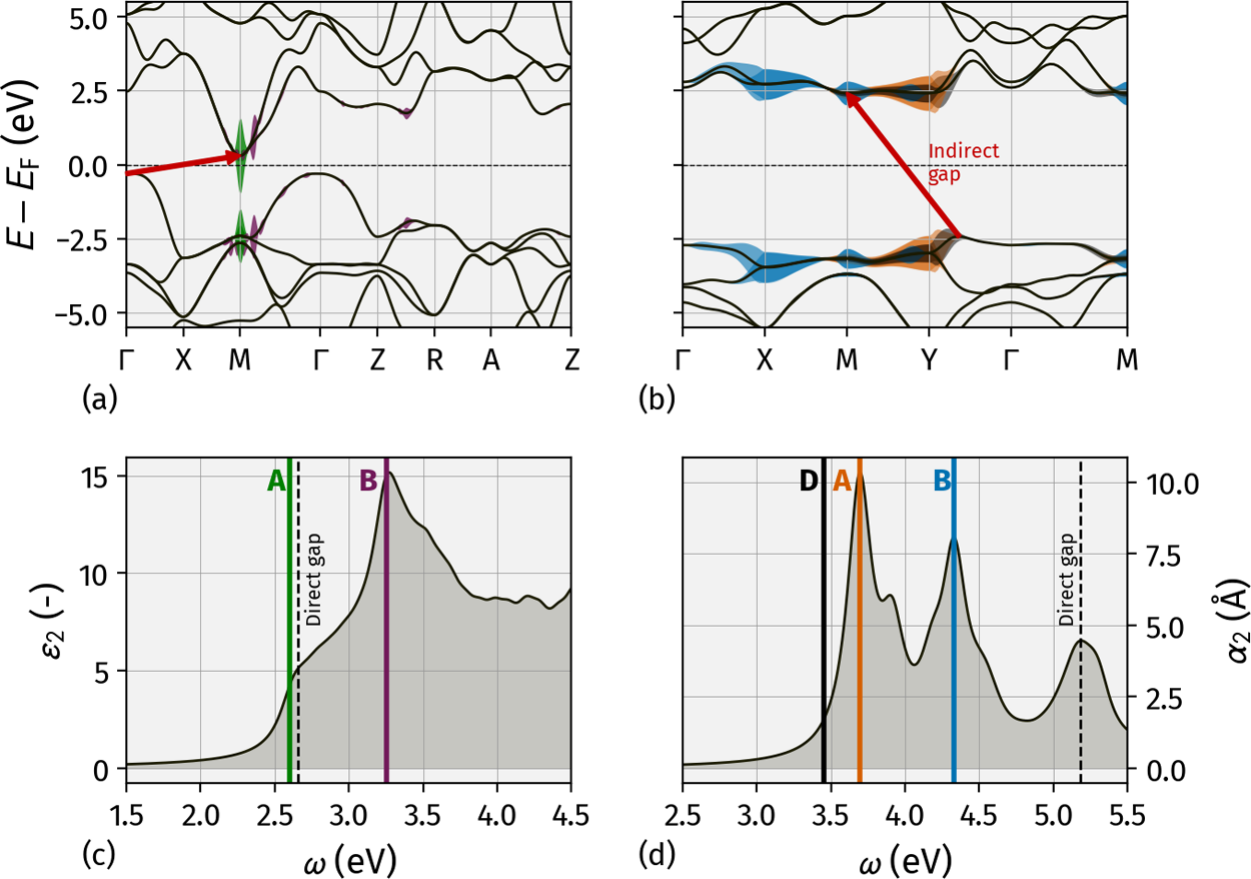
Upper panels: QP band structures of SnO bulk (a) and ML (b) aligned to the Fermi energy. The areas in color around the top valence bands and the bottom conduction bands highlight the electronic transitions contributing to the exciton states, with corresponding colors matching those in panels (c) and (d). Lower panels: absorption spectra of SnO bulk and ML. The solid vertical lines mark the energy of selected exciton states whose main electronic transitions are highlighted in panels (a) and (b) with corresponding colors.

The QP band structures of the SnO bulk and ML, aligned to the Fermi energy, are reported in Figure 3a,b, respectively. In the bulk system, panel (a), the inclusion of the QP corrections results in an indirect gap of 0.61 eV between the Γ and the M point, in line with the experiment^1^ and previous calculations.^59^ In the ML, the indirect bandgap increases to 4.85 eV, between tVB at a point near Y along the Y̅Γ̅ direction and the bCB at M, panel (b). The substantial increase in the indirect gap magnitude when passing from bulk to ML has been related to the smaller dispersion of the band edges in the ML due to the absence of interlayer interactions.^57,60^

The absorption spectra, computed by solving the BSE, are shown in the bottom panels of Figure 3. For bulk SnO, the imaginary part of the macroscopic dielectric function, ε_2_, shown in panel (a), corresponds to the absorption of light with linear polarization along the [1,1,1] direction. Bulk SnO displays an evident absorption peak, labeled B, above 3 eV and a long tail extending to nearly 2 eV. Peak B is mainly the result of electronic transitions between tVB and bCB at *k*-points along the M̅Γ̅ direction. Additionally, it receives minor contributions from transitions between Z and R, as marked in purple in panel (c). The lower absorption onset is due to the lowest-energy, bright exciton state, A, at 2.34 eV, whose dominant electronic transitions, marked in green in panel (a), occur between tVB and bCB at the M point.

**Figure 4:**
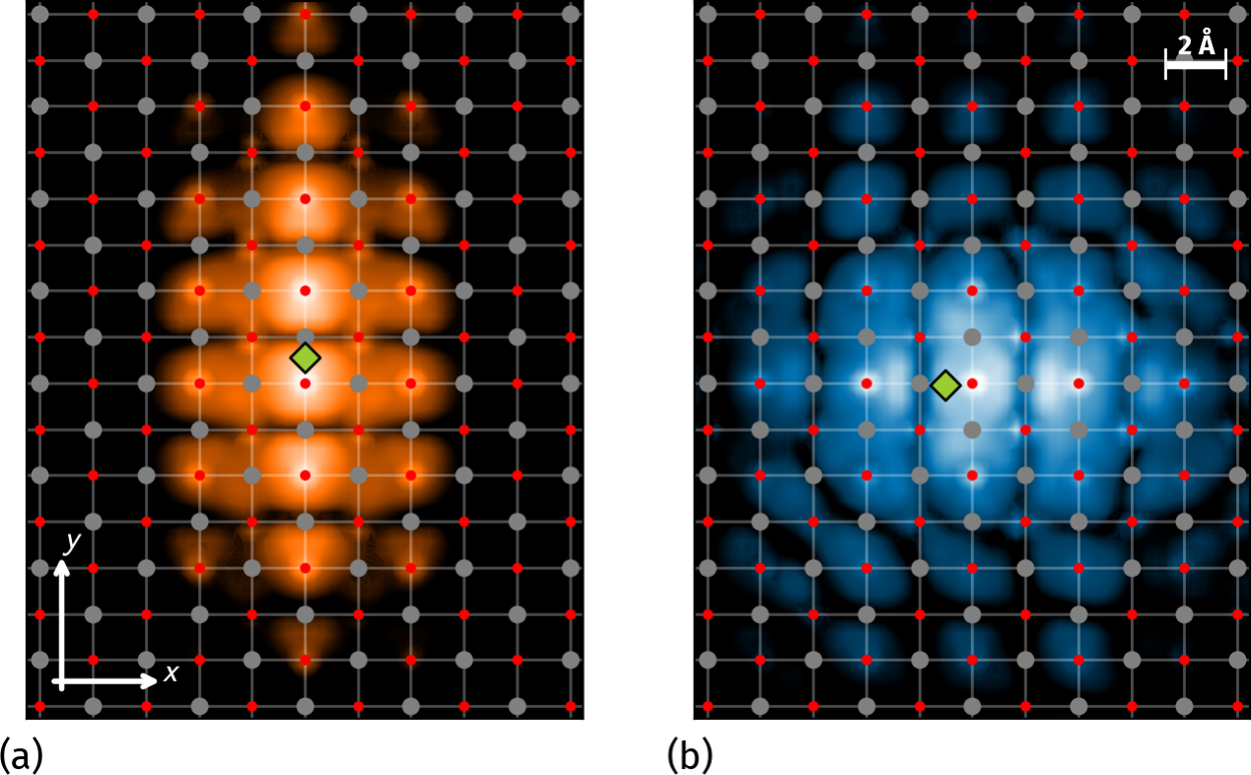
Squared moduli of the SnO ML exciton wave functions for state A, in panel (a), and B, in panel (b). The green diamonds mark the position of the hole. Gray and red dots indicate Sn and O atoms, respectively.

In the case of SnO ML, absorption is physically described by the imaginary part of the macroscopic polarizability, α_2_, in place of the dielectric function, which is ill-defined for 2D systems.^47,61^ The spectrum in Figure 3d corresponds to the absorption of light with in-plane linear polarization along the [1,1,0] direction. In contrast to the bulk system, the lowest-energy state is now a dark exciton, D, mainly due to transitions around the Y point, marked by the black areas in Figure 3b. Electronic transitions around Y between tVB and bCB, marked in orange in panel (b), give origin to the bright state A, at 3.70 eV, responsible for the absorption onset of the material in panel (d). A second absorption peak appears in the spectrum at 4.33 eV, originating from exciton B, whose electronic transitions mainly occur between tVB and bCB around X, blue regions in panel (b). Comparing Figure 3b,d with Figure 2b, it is evident that the dominant features of ML absorption are associated with exciton states involving electronic transitions at the two inequivalent points X and Y, where the electron wave functions predominantly project onto p_*x*_ and p_*y*_ atomic orbitals, respectively. This is reflected in Figure 4, where panels (a) and (b) display the squared moduli of the wave functions for excitons A and B, respectively. The position of the hole (indicated by the green diamond) is fixed in a region where the electron density of the tVB is at its maximum. Exciton A, in panel (a), exhibits marked anisotropy along the *y* axis and is predominantly composed of p_*y*_ orbitals of both Sn and O atoms. Conversely, exciton B, in panel (b), displays a dominant p_*x*_ character and extends along the *x* direction. Given the different orbital contributions, we expect the absorption behavior to change with the light polarization angle, φ. The dominant contribution of p_*x*_ orbitals around the X point should result in a net orientation of electronic dipoles along the *x* axis and, consequently, favor the absorption of light polarized along *x*, with φ = 0. Conversely, the prevalence of p_*y*_ orbitals at Y should enhance the absorption of *y*-polarized light, φ = 90°, and suppress the absorption of orthogonal polarization.

We can make more sense of this mechanism by analyzing the group representations of the states involved in the electronic transitions at X and Y. For simplicity, we consider the symmorphic subgroup *pmm2* of the full layer group *pmmn*. We refer the reader to refs (62, 63) for the group theory background relevant to this analysis. The group of the wavevector at both X and Y is *mm2*, or *C*_2*v*_, so that each electronic state, at both *k*-points, corresponds to one of its irreducible representations. The dipole operator **d** of in-plane polarized light transforms as *B*_2_[*x*] ⊕ *B*_1_[*y*]. To yield a nonzero result, the dipole matrix element ⟨*c*_**k**_|**d**|*v*_**k**_⟩, with |*v*_**k**_⟩, |*c*_**k**_⟩ valence and conduction band states at **k**, must include at least one component transforming as the fully symmetric representation *A*_1_. At the X point, the dipole transitions contributing to peak B connect tVB and bCB, which are doubly degenerate, and both transform as *A*_1_ ⊕ *B*_2_. Therefore, the only nonzero contributions to the dipole matrix element result from the coupling with *x*-polarized light as follows: (*A*_1_ ⊕ *B*_2_) ⊗ *B*_2_[*x*] ⊗ (*A*_1_ ⊕ *B*_2_). Conversely, at Y, tVB and bCB transform as *A*_1_ ⊕ *B*_1_, so that the only nonvanishing dipole matrix elements arise from *y*-polarized light as (*A*_1_ ⊕ *B*_1_) ⊗ *B*_1_[*y*] ⊗ (*A*_1_ ⊕ *B*_1_). We conclude that electronic transitions at X and Y, respectively, responsible for the most prominent absorption peaks B and A, couple with orthogonal polarizations. Specifically, transitions at X absorb *x*-polarized light, while those at Y preferentially absorb *y*-polarized light.

**Figure 5:**
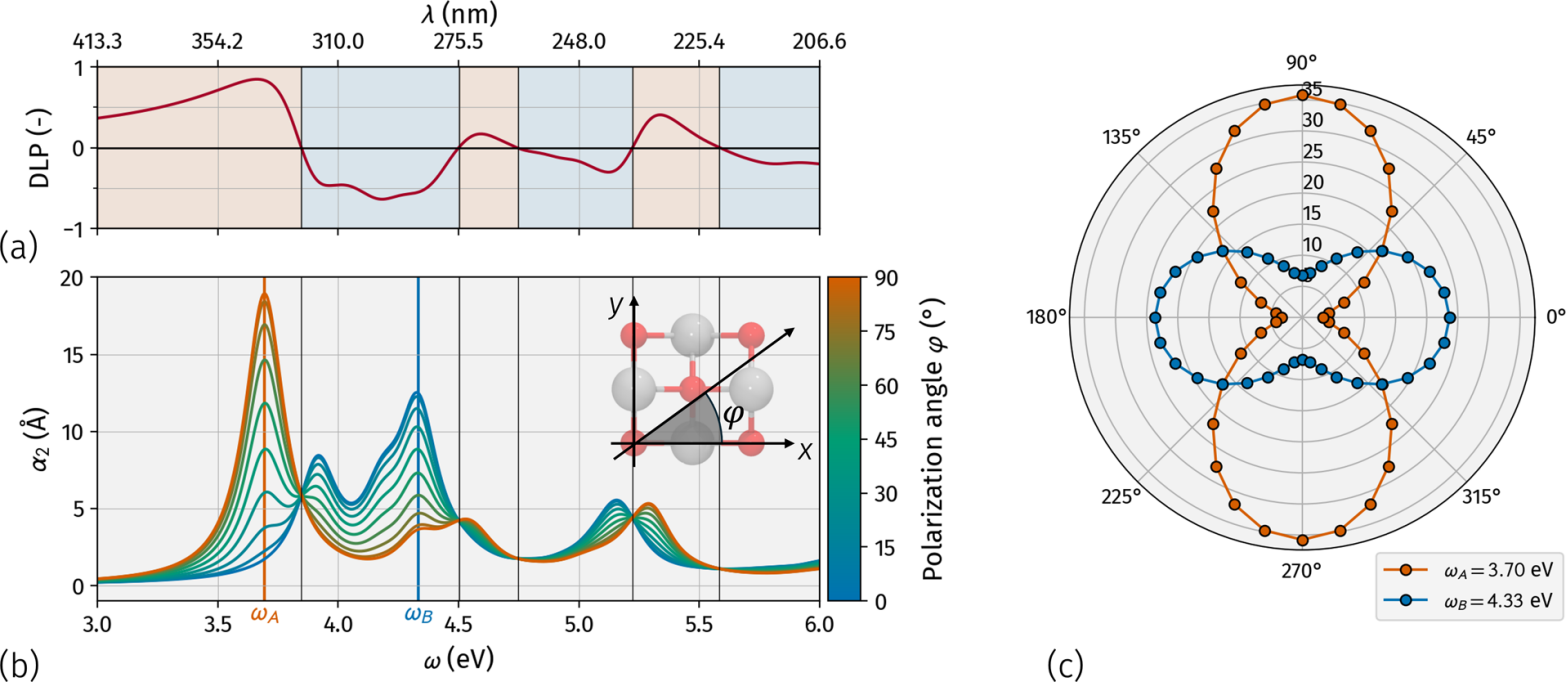
Linear dichroism inversion in SnO ML. (a) Degree of linear polarization (DLP), (b) absorption spectra for different polarization angles φ, as defined in the inset, and (c) polar plot of absorption at ω_A_ (orange curve) and ω_B_ (blue curve).

This behavior is explicitly verified in Figure 5, where, in panel (b), we plot the absorption spectra for different polarization angles of 0 ≤ φ ≤ 90°. For φ = 0, light polarized along *x*, the A peak at ω_A_ = 3.70 eV disappears, the absorption onset blueshifts, and B becomes the most intense absorption peak. As the polarization angle increases, the intensity of the A peak grows, while that of the B peak decreases. For φ = 90°, the B peak is significantly reduced, with the A peak dominating the spectrum. Since peaks A and B occur at different energies, we have a different response to linearly polarized light at varying frequencies. This is evident in Figure 5c, where the polar plot shows the absorbance as a function of the polarization angle φ at two different frequencies, ω_A_ and ω_B_ (orange and blue curves, respectively), corresponding to peaks A and B. The two-lobe curves clearly present absorption maxima for orthogonal polarizations. SnO ML then shows LDI, selectively absorbing orthogonal polarizations at different frequencies. In Figure 5a, in correspondence with the absorption spectra, we report the degree of linear polarization (DLP), defined as DLP = (α_2,*y*_ – α_2,*x*_)/(α_2,*x*_ + α_2,*y*_), where α_2,*x*_, and α_2,*y*_ are the imaginary part of the macroscopic polarizability for light polarized along the *x* and *y* axes, respectively. The DLP can vary from −1 (indicating complete absorption of *x*-polarized light and total transmission of *y*-polarized light), through 0 (showing equivalent absorption for both polarizations), to +1 (total absorption of *y*-polarized light and complete transmission of *x*-polarized light). The LDI shown in the polar plot in panel (c) corresponds to the change in sign of the DLP at around 3.8 eV, panel (a). Here, the DLP shifts from almost +1 to below −0.5. However, the DLP crosses zero four additional times between 4 and 6 eV, indicating multiple reversals in crystal dichroism. Overall, from the absorption onset up to 6 eV, corresponding to the wavelengths from approximately 200 to 400 nm, we identify six frequency windows where SnO ML preferentially absorbs either one or the orthogonal linear polarization. For comparison, recently synthesized SiP shows a single LDI in the 400 to 900 nm range,^36^ while PdPS exhibits four LDIs between 200 and 700 nm.^37^ These findings indicate SnO ML as a remarkable candidate for experimental studies investigating its potential to differentiate among various frequency ranges in the UV spectrum.

## 4 Conclusions

Our analysis examines the impact of the reduced spatial symmetry of the ferroelastic phase of SnO ML on its electronic and optical properties. The transition from bulk to ferroelastic ML involves a shift from a tetragonal system to an orthorhombic one. The resulting inequivalence of the *x* and *y* spatial directions is reflected in the different orbital characters of the band edges states along the orthogonal M̅X̅ and M̅Y̅ directions in the BZ: around the X and Y points, the electronic states of tVB and bCB primarily involve p_*x*_ and p_*y*_ atomic orbitals, respectively. The investigation of the optical absorption and excitonic properties reveals that electronic transitions along M̅X̅ and M̅Y̅ in the BZ are responsible for the main features of the spectrum. The first peak results from transitions around Y, followed by a second structure originating from transitions around X. Hence, SnO ML presents LDI, preferentially absorbing either one or the orthogonal polarization, depending on the light frequency. The DLP analysis further identifies multiple reversals of polarization direction across wavelengths from 200 to 400 nm, suggesting that ML SnO could join the group of low-symmetry 2D materials with unique optical responses to polarized light. Given its absorption in UV, its cost-effectiveness, and relatively easy exfoliation, ML SnO emerges as a promising semiconductor for polarization-sensitive UV photodetection at the nanoscale.

## Supporting Information Available

Convergence of DFT, GW, and BSE calculations, optimized structures of SnO bulk, tri-, bi-, and monolayers, pDOS and *k*-resolved pDOS of SnO bulk and ML, and phonon dispersions of SnO bulk and ML (PDF)

## References

[ref1] Ogo Y.; Hiramatsu H.; Nomura K.; Yanagi H.; Kamiya T.; Hirano M.; Hosono H. p-channel thin-film transistor using p-type oxide semiconductor, SnO. Appl. Phys. Lett. 2008, 93, 032113 10.1063/1.2964197.

[ref2] Caraveo-Frescas J. A.; Nayak P. K.; Al-Jawhari H. A.; Granato D. B.; Schwingenschlögl U.; Alshareef H. N. Record Mobility in Transparent p-Type Tin Monoxide Films and Devices by Phase Engineering. ACS Nano 2013, 7, 5160–5167. 10.1021/nn400852r. 23668750

[ref3] Wang Z.; Nayak P. K.; Caraveo-Frescas J. A.; Alshareef H. N. Recent Developments in p-Type Oxide Semiconductor Materials and Devices. Adv. Mater. 2016, 28, 3831–3892. 10.1002/adma.201503080. 26879813

[ref4] Liu A.; Zhu H.; Noh Y.-Y. Solution-processed inorganic p-channel transistors: Recent advances and perspectives. Mater. Sci. Eng., R 2019, 135, 85–100. 10.1016/j.mser.2018.11.001.

[ref5] Minohara M.; Hase I.; Aiura Y. Characteristic Electronic Structure of SnO Film Showing High Hole Mobility. J. Phys. Chem. Lett. 2022, 13, 1165–1171. 10.1021/acs.jpclett.1c04182. 35084204

[ref6] Liang L. Y.; Liu Z. M.; Cao H. T.; Pan X. Q. Microstructural, Optical, and Electrical Properties of SnO Thin Films Prepared on Quartz *via* a Two-Step Method. ACS Appl. Mater. Interfaces 2010, 2, 1060–1065. 10.1021/am900838z. 20423126

[ref7] Guo Y.; Ma L.; Mao K.; Ju M.; Bai Y.; Zhao J.; Zeng X. C. Eighteen functional monolayer metal oxides: wide bandgap semiconductors with superior oxidation resistance and ultrahigh carrier mobility. Nanoscale Horiz. 2019, 4, 592–600. 10.1039/C8NH00273H. 32254160

[ref8] Kim T.; Kim J.-K.; Yoo B.; Xu H.; Yim S.; Kim S.-H.; Yu H.-Y.; Jeong J. K. Improved switching characteristics of p-type tin monoxide field-effect transistors through Schottky energy barrier engineering. J. Mater. Chem. C 2020, 8, 201–208. 10.1039/C9TC04345D.

[ref9] Huang C.-H.; Tang Y.; Yang T.-Y.; Chueh Y.-L.; Nomura K. Atomically Thin Tin Monoxide-Based p-Channel Thin-Film Transistor and a Low-Power Complementary Inverter. ACS Appl. Mater. Interfaces 2021, 13, 52783–52792. 10.1021/acsami.1c15990. 34719921

[ref10] Kim T.; Lee H.; Kim S. E.; Kim J.-K.; Jeong J. K. High mobility p-channel tin monoxide thin-film transistors with hysteresis-free like behavior. Appl. Phys. Lett. 2022, 121, 142101 10.1063/5.0115893.

[ref11] Hota M. K.; Caraveo-Frescas J. A.; McLachlan M. A.; Alshareef H. N. Electroforming-free resistive switching memory effect in transparent p-type tin monoxide. Appl. Phys. Lett. 2014, 104, 152104 10.1063/1.4870405.

[ref12] Hota M. K.; Hedhili M. N.; Wang Q.; Melnikov V. A.; Mohammed O. F.; Alshareef H. N. Nanoscale Cross-Point Resistive Switching Memory Comprising p-Type SnO Bilayers. Adv. Electron. Mater. 2015, 1, 1400035 10.1002/aelm.201400035.

[ref13] Sinha A. K.; Manna P. K.; Pradhan M.; Mondal C.; Yusuf S. M.; Pal T. Tin oxide with a p–n heterojunction ensures both UV and visible light photocatalytic activity. RSC Adv. 2014, 4, 208–211. 10.1039/C3RA42740D.

[ref14] Liang L.; Sun Y.; Lei F.; Gao S.; Xie Y. Free-floating ultrathin tin monoxide sheets for solar-driven photoelectrochemical water splitting. J. Mater. Chem. A 2014, 2, 10647–10653. 10.1039/c4ta01659a.

[ref15] Zhang F.; Zhu J.; Zhang D.; Schwingenschlögl U.; Alshareef H. N. Two-Dimensional SnO Anodes with a Tunable Number of Atomic Layers for Sodium Ion Batteries. Nano Lett. 2017, 17, 1302–1311. 10.1021/acs.nanolett.6b05280. 28098459

[ref16] Ren Q.; Zhang X.; Guo Y.; Xu M.; Zhu H.; Yun J.; Zhao W.; Zhang Z.; Wang Y. Shape-controlled SnO and their improved properties in the field of gas sensor, photocatalysis, and lithium-ion battery. Sens. Actuators, B 2022, 372, 132622 10.1016/j.snb.2022.132622.

[ref17] Wan W.; Ge Y.; Liu Y. Strong phonon anharmonicity and low thermal conductivity of monolayer tin oxides driven by lone-pair electrons. Appl. Phys. Lett. 2019, 114, 031901 10.1063/1.5063560.

[ref18] Bishop T. B.; Farmer E. E.; Sharmin A.; Pacheco-Sanjuan A.; Darancet P.; Barraza-Lopez S. Quantum Paraelastic Two-Dimensional Materials. Phys. Rev. Lett. 2019, 122, 015703 10.1103/PhysRevLett.122.015703. 31012714

[ref19] Gao P.; Britson J.; Nelson C. T.; Jokisaari J. R.; Duan C.; Trassin M.; Baek S.-H.; Guo H.; Li L.; Wang Y.; Chu Y.-H.; Minor A. M.; Eom C.-B.; Ramesh R.; Chen L.-Q.; Pan X. Ferroelastic domain switching dynamics under electrical and mechanical excitations. Nat. Commun. 2014, 5, 3801 10.1038/ncomms4801. 24787035

[ref20] Xue F.; Li Y.; Gu Y.; Zhang J.; Chen L.-Q. Strain phase separation: Formation of ferroelastic domain structures. Phys. Rev. B 2016, 94, 220101 10.1103/PhysRevB.94.220101.

[ref21] Chen Q.; Zhang Y.; Zheng T.; Liu Z.; Wu L.; Wang Z.; Li J. Polarization detection in deep-ultraviolet light with monoclinic gallium oxide nanobelts. Nanoscale Adv. 2020, 2, 2705–2712. 10.1039/D0NA00364F. 36132414 PMC9419289

[ref22] Zhang Y.; Wang Z.; Xing F. Enhancement of polarization response in UVA and UVC wavelength with integrated sub-wavelength metal-grids. Microelectron. Eng. 2021, 242–243, 111555 10.1016/j.mee.2021.111555.

[ref23] Kim D.; Park K.; Lee J. H.; Kwon I. S.; Kwak I. H.; Park J. Anisotropic 2D SiAs for High-Performance UV–Visible Photodetectors. Small 2021, 17, 2006310 10.1002/smll.202006310. 33590682

[ref24] Xu Z.; Weng W.; Li Y.; Liu X.; Yang T.; Li M.; Huang X.; Luo J.; Sun Z. 3D-to-2D Dimensional Reduction for Exploiting a Multilayered Perovskite Ferroelectric toward Polarized-Light Detection in the Solar-Blind Ultraviolet Region. Angew. Chem., Int. Ed. 2020, 59, 21693–21697. 10.1002/anie.202009329. 32798285

[ref25] Barraza-Lopez S.; Xia F.; Zhu W.; Wang H. Beyond Graphene: Low-Symmetry and Anisotropic 2D Materials. J. Appl. Phys. 2020, 128, 140401 10.1063/5.0030751.

[ref26] Xia F.; Wang H.; Jia Y. Rediscovering black phosphorus as an anisotropic layered material for optoelectronics and electronics. Nat. Commun. 2014, 5, 4458 10.1038/ncomms5458. 25041752

[ref27] Qiao J.; Kong X.; Hu Z.-X.; Yang F.; Ji W. High-mobility transport anisotropy and linear dichroism in few-layer black phosphorus. Nat. Commun. 2014, 5, 4475 10.1038/ncomms5475. 25042376 PMC4109013

[ref28] Yuan H.; Liu X.; Afshinmanesh F.; Li W.; Xu G.; Sun J.; Lian B.; Curto A. G.; Ye G.; Hikita Y.; Shen Z.; Zhang S.-C.; Chen X.; Brongersma M.; Hwang H. Y.; Cui Y. Polarization-sensitive broadband photodetector using a black phosphorus vertical p–n junction. Nat. Nanotechnol. 2015, 10, 707–713. 10.1038/nnano.2015.112. 26030655

[ref29] Yang Y.; Liu S.-C.; Yang W.; Li Z.; Wang Y.; Wang X.; Zhang S.; Zhang Y.; Long M.; Zhang G.; Xue D.-J.; Hu J.-S.; Wan L.-J. Air-Stable In-Plane Anisotropic GeSe2 for Highly Polarization-Sensitive Photodetection in Short Wave Region. J. Am. Chem. Soc. 2018, 140, 4150–4156. 10.1021/jacs.8b01234. 29494139

[ref30] Zhou Z.; Cui Y.; Tan P.-H.; Liu X.; Wei Z. Optical and electrical properties of two-dimensional anisotropic materials. J. Semicond. 2019, 40, 061001 10.1088/1674-4926/40/6/061001.

[ref31] Suk S. H.; Seo S. B.; Cho Y. S.; Wang J.; Sim S. Nanophotonics 2024, 13, 107–154. 10.1515/nanoph-2023-0639. 39635300 PMC11501201

[ref32] Wu J.; Cong X.; Niu S.; Liu F.; Zhao H.; Du Z.; Ravichandran J.; Tan P.-H.; Wang H. Linear Dichroism Conversion in Quasi-1D Perovskite Chalcogenide. Adv. Mater. 2019, 31, 1902118 10.1002/adma.201902118. 31237378

[ref33] Zhang H.; Li Y.; Hu X.; Xu J.; Chen L.; Li G.; Yin S.; Chen J.; Tan C.; Kan X.; Li L. In-plane anisotropic 2D CrPS4 for promising polarization-sensitive photodetection. Appl. Phys. Lett. 2021, 119, 171102 10.1063/5.0066143.

[ref34] Wang X.; Li Y.; Huang L.; Jiang X.-W.; Jiang L.; Dong H.; Wei Z.; Li J.; Hu W. Short-Wave Near-Infrared Linear Dichroism of Two-Dimensional Germanium Selenide. J. Am. Chem. Soc. 2017, 139, 14976–14982. 10.1021/jacs.7b06314. 28926248

[ref35] Yu J.; Kuang X.; Gao Y.; Wang Y.; Chen K.; Ding Z.; Liu J.; Cong C.; He J.; Liu Z.; Liu Y. Direct Observation of the Linear Dichroism Transition in Two-Dimensional Palladium Diselenide. Nano Lett. 2020, 20, 1172–1182. 10.1021/acs.nanolett.9b04598. 31944114

[ref36] Xie X.; Ding J.; Wu B.; Zheng H.; Li S.; Wang C.-T.; He J.; Liu Z.; Wang J.-T.; Duan J.-A.; Liu Y. Observation of optical anisotropy and a linear dichroism transition in layered silicon phosphide. Nanoscale 2023, 15, 12388–12397. 10.1039/D3NR01765F. 37455620

[ref37] Li G.; Chen Z.; Zhang H.; Yu M.; Zhang H.; Chen J.; Wang Z.; Yin S.; Lin W.; Gong P.; Zeng L.; Zhu X.; Wei W.; Tian M.; Li L. Abnormal linear dichroism transition in two-dimensional PdPS. Nanoscale 2022, 14, 14129–14134. 10.1039/D2NR03587A. 36111459

[ref38] Wu D.; Guo J.; Du J.; Xia C.; Zeng L.; Tian Y.; Shi Z.; Tian Y.; Li X. J.; Tsang Y. H.; Jie J. Highly Polarization-Sensitive, Broadband, Self-Powered Photodetector Based on Graphene/PdSe2/Germanium Heterojunction. ACS Nano 2019, 13, 9907–9917. 10.1021/acsnano.9b03994. 31361122

[ref39] Kumar A.; Khan M. A.; Kumar M. Recent advances in UV photodetectors based on 2D materials: a review. J. Phys. D: Appl. Phys. 2022, 55, 133002 10.1088/1361-6463/ac33d7.

[ref40] Giannozzi P.; Baroni S.; Bonini N.; Calandra M.; Car R.; Cavazzoni C.; Ceresoli D.; Chiarotti G. L.; Cococcioni M.; Dabo I.; Corso A. D.; de Gironcoli S.; Fabris S.; Fratesi G.; Gebauer R.; Gerstmann U.; Gougoussis C.; Kokalj A.; Lazzeri M.; Martin-Samos L.; Marzari N.; Mauri F.; Mazzarello R.; Paolini S.; Pasquarello A.; Paulatto L.; Sbraccia C.; Scandolo S.; Sclauzero G.; Seitsonen A. P.; Smogunov A.; Umari P.; Wentzcovitch R. M. QUANTUM ESPRESSO: a modular and open-source software project for quantum simulations of materials. J. Phys.: Condens. Matter 2009, 21, 395502 10.1088/0953-8984/21/39/395502. 21832390

[ref41] Giannozzi P.; Andreussi O.; Brumme T.; Bunau O.; Nardelli M. B.; Calandra M.; Car R.; Cavazzoni C.; Ceresoli D.; Cococcioni M.; Colonna N.; Carnimeo I.; Corso A. D.; de Gironcoli S.; Delugas P.; DiStasio R. A.; Ferretti A.; Floris A.; Fratesi G.; Fugallo G.; Gebauer R.; Gerstmann U.; Giustino F.; Gorni T.; Jia J.; Kawamura M.; Ko H.-Y.; Kokalj A.; Küçükbenli E.; Lazzeri M.; Marsili M.; Marzari N.; Mauri F.; Nguyen N. L.; Nguyen H.-V.; de-la Roza A. O.; Paulatto L.; Poncé S.; Rocca D.; Sabatini R.; Santra B.; Schlipf M.; Seitsonen A. P.; Smogunov A.; Timrov I.; Thonhauser T.; Umari P.; Vast N.; Wu X.; Baroni S. Advanced capabilities for materials modelling with Quantum ESPRESSO. J. Phys.: Condens. Matter 2017, 29, 465901 10.1088/1361-648X/aa8f79. 29064822

[ref42] Carnimeo I.; Affinito F.; Baroni S.; Baseggio O.; Bellentani L.; Bertossa R.; Delugas P. D.; Ruffino F. F.; Orlandini S.; Spiga F.; Giannozzi P. Quantum ESPRESSO: One Further Step toward the Exascale. J. Chem. Theory Comput. 2023, 19, 6992–7006. 10.1021/acs.jctc.3c00249. 37523670 PMC10601483

[ref43] Perdew J. P.; Burke K.; Ernzerhof M. Generalized Gradient Approximation Made Simple. Phys. Rev. Lett. 1996, 77, 3865–3868. 10.1103/PhysRevLett.77.3865. 10062328

[ref44] Grimme S.; Antony J.; Ehrlich S.; Krieg H. A consistent and accurate ab initio parametrization of density functional dispersion correction (DFT-D) for the 94 elements H-Pu. J. Chem. Phys. 2010, 132, 154104 10.1063/1.3382344. 20423165

[ref45] Troullier N.; Martins J. L. Efficient pseudopotentials for plane-wave calculations. Phys. Rev. B 1991, 43, 1993–2006. 10.1103/PhysRevB.43.1993. 9997467

[ref46] Monkhorst H. J.; Pack J. D. Special points for Brillouin-zone integrations. Phys. Rev. B 1976, 13, 5188–5192. 10.1103/PhysRevB.13.5188.

[ref47] Marini A.; Hogan C.; Grüning M.; Varsano D. yambo: An ab initio tool for excited state calculations. Comput. Phys. Commun. 2009, 180, 1392–1403. 10.1016/j.cpc.2009.02.003.

[ref48] Sangalli D.; Ferretti A.; Miranda H.; Attaccalite C.; Marri I.; Cannuccia E.; Melo P.; Marsili M.; Paleari F.; Marrazzo A.; Prandini G.; Bonfà P.; Atambo M. O.; Affinito F.; Palummo M.; Molina-Sánchez A.; Hogan C.; Grüning M.; Varsano D.; Marini A. Many-body perturbation theory calculations using the yambo code. J. Phys.: Condens. Matter 2019, 31, 325902 10.1088/1361-648X/ab15d0. 30943462

[ref49] Rojas H. N.; Godby R. W.; Needs R. J. Space-Time Method for Ab Initio Calculations of Self-Energies and Dielectric Response Functions of Solids. Phys. Rev. Lett. 1995, 74, 1827–1830. 10.1103/PhysRevLett.74.1827. 10057767

[ref50] Guandalini A.; D’Amico P.; Ferretti A.; Varsano D. Efficient GW calculations in two dimensional materials through a stochastic integration of the screened potential. npj Comput. Mater. 2023, 9, 44 10.1038/s41524-023-00989-7.

[ref51] Rojas H. N.; Godby R. W.; Needs R. J. Space-Time Method for Ab Initio Calculations of Self-Energies and Dielectric Response Functions of Solids. Phys. Rev. Lett. 1995, 74, 1827–1830. 10.1103/PhysRevLett.74.1827. 10057767

[ref52] Pannetier J.; Denes G. Tin(II) oxide: structure refinement and thermal expansion. Acta Crystallogr., Sect. B: Struct. Sci. 1980, 36, 2763–2765. 10.1107/S0567740880009934.

[ref53] Moreno M. S.; Mercader R. C. Mössbauer study of SnO lattice dynamics. Phys. Rev. B 1994, 50, 9875–9881. 10.1103/PhysRevB.50.9875. 9975068

[ref54] Walsh A.; Watson G. W. Electronic structures of rocksalt, litharge, and herzenbergite SnO by density functional theory. Phys. Rev. B 2004, 70, 235114 10.1103/PhysRevB.70.235114.

[ref55] Walsh A.; Watson G. W. Influence of the Anion on Lone Pair Formation in Sn(II) Monochalcogenides: A DFT Study. J. Phys. Chem. B 2005, 109, 18868–18875. 10.1021/jp051822r. 16853428

[ref56] Walsh A.; Payne D. J.; Egdell R. G.; Watson G. W. Stereochemistry of post-transition metal oxides: revision of the classical lone pair model. Chem. Soc. Rev. 2011, 40, 4455–4463. 10.1039/c1cs15098g. 21666920

[ref57] Zhou W.; Umezawa N. Band gap engineering of bulk and nanosheet SnO: an insight into the interlayer Sn–Sn lone pair interactions. Phys. Chem. Chem. Phys. 2015, 17, 17816–17820. 10.1039/C5CP02255J. 26088037

[ref58] Hu Y.; Schlom D.; Datta S.; Cho K. Interlayer Engineering of Band Gap and Hole Mobility in p-Type Oxide SnO. ACS Appl. Mater. Interfaces 2022, 14, 25670–25679. 10.1021/acsami.2c03554. 35609177

[ref59] Wu Y.; Tang Z.; Cruz G. J.; Yang Y.; Zhang W.; Ren W.; Zhang P. Exploiting the stereoelectronic effects for selective tuning of band edge states of α-SnO: *GW* quasiparticle calculations. Phys. Rev. B 2022, 106, 085201 10.1103/PhysRevB.106.085201.

[ref60] Wanzhong L.; Jian S.; Chong D. Layer-dependent electronic and optical properties of tin monoxide: a potential candidate in photovoltaic applications. Phys. Chem. Chem. Phys. 2022, 24, 7611–7616. 10.1039/D1CP05305A. 35311866

[ref61] Cudazzo P.; Tokatly I. V.; Rubio A. Dielectric screening in two-dimensional insulators: Implications for excitonic and impurity states in graphane. Phys. Rev. B 2011, 84, 085406 10.1103/PhysRevB.84.085406.

[ref62] Dresselhaus M. S.; Dresselhaus G.; Jorio A. Group theory: application to the physics of condensed matter; Springer Science & Business Media, 2007.

[ref63] Ludwig W.; Falter C. Symmetries in Physics: Group Theory Applied to Physical Problems; Springer Series in Solid-State Sciences; Springer: Berlin Heidelberg, 2012.

