# Supplementary material for: Multiple Linear Dichroism Inversions in SnO Monolayers for Polarization-Sensitive UV Photodetection: An Ab Initio Investigation
Source: ACS Appl Nano Mater. 2025 Jan 30;8(5):2374–81. doi: 10.1021/acsanm.4c06552 (PMC11812053; doi:10.1021/acsanm.4c06552)
Supplement: Supplementary file 1 — an4c06552_si_001.pdf [file an4c06552_si_001.pdf]

Supporting Information

Multiple Linear Dichroism Inversions in SnO Monolayers for Polarization-Sensitive UV Photodetection: an ab Initio Investigation

Michele Re Fiorentin[1,*], Francesca Risplendi[1], Maurizia Palummo[2] and Giancarlo Cicero[1]

[1]*Department of Applied Science and Technology, Politecnico di Torino corso Duca degli Abruzzi 24, 10129 Torino, Italy*

[2]*Dipartimento di Fisica and INFN, Università di Roma "Tor Vergata", via della Ricerca Scientifica 1, 00133, Roma, Italy*

S1 DFT calculations

S1.1 Convergence

The convergence of DFT calculations with respect to the plane-wave energy cutoff, E_{cut} and the k-point grid for bulk and ML is reported in Fig. S1. In all cases we converge the variations of system total energy per atom, ΔE^{tot}/atom, to within 5 meV/atom.

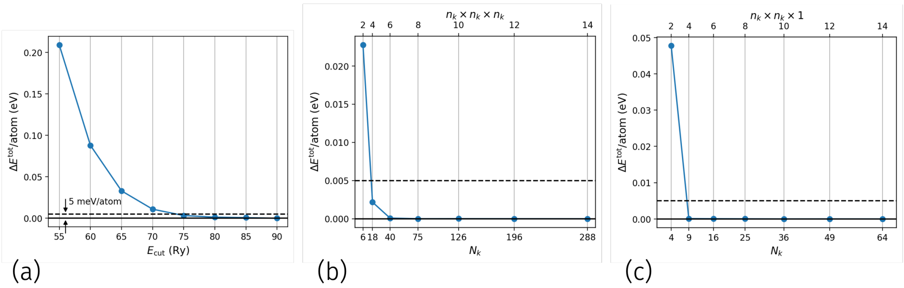

Figure S1: Convergence of DFT calculations with respect to the plane-wave energy cutoff (a) and the number of k-points for SnO bulk (b) and ML (c).

S1.2 Optimized structures

Here we report the structural data of the optimized SnO bulk, trilayer, bilayer and monolayer. Data are reported as CIF file incorporating the explicit group analysis provided by the FINDSYM interface [1, 2].

S1.2.1 Bulk

```
    # CIF file created by FINDSYM, version 7.1.3

data_findsym-output
_audit_creation_method FINDSYM
```

*michele.refiorentin@polito.it

```
_cell_length_a     3.8310300000
_cell_length_b     3.8310300000
_cell_length_c     4.7845600000
_cell_angle_alpha  90.0000000000
_cell_angle_beta   90.0000000000
_cell_angle_gamma  90.0000000000
_cell_volume       70.2219864814

_symmetry_space_group_name_H-M "P 4/n 21/m 2/m (origin choice 2)"
_symmetry_Int_Tables_number 129
_space_group.reference_setting '129:-P 4a 2a'
_space_group.transform_Pp_abc a,b,c;0,0,0

loop_
_space_group_symop_id
_space_group_symop_operation_xyz
1 x,y,z
2 x+1/2,-y,-z
3 -x,y+1/2,-z
4 -x+1/2,-y+1/2,z
5 -y,-x,-z
6 -y+1/2,x,z
7 y,-x+1/2,z
8 y+1/2,x+1/2,-z
9 -x,-y,-z
10 -x+1/2,y,z
11 x,-y+1/2,z
12 x+1/2,y+1/2,-z
13 y,x,z
14 y+1/2,-x,-z
15 -y,x+1/2,-z
16 -y+1/2,-x+1/2,z

loop_
_atom_type_symbol
Sn
O

loop_
_atom_site_label
_atom_site_type_symbol
_atom_site_symmetry_multiplicity
_atom_site_Wyckoff_symbol
_atom_site_fract_x
_atom_site_fract_y
```

```
_atom_site_fract_z
_atom_site_occupancy
_atom_site_fract_symmform
Sn1 Sn   2 c  0.2500000000  0.2500000000  0.2403250000  1.0000000000 0,0,Dz
O1  O    2 a  0.7500000000  0.2500000000  0.0000000000  1.0000000000 0,0,0

# end of cif
```

S1.2.2 Trilayer

```
# CIF file created by FINDSYM, version 7.1.3

data_findsym-output
_audit_creation_method FINDSYM

_cell_length_a     3.8241900000
_cell_length_b     3.8241900000
_cell_length_c     24.4391000000
_cell_angle_alpha  90.0000000000
_cell_angle_beta   90.0000000000
_cell_angle_gamma  90.0000000000
_cell_volume       357.4078865888

_symmetry_space_group_name_H-M "P 4/n 21/m 2/m (origin choice 2)"
_symmetry_Int_Tables_number 129
_space_group.reference_setting '129:-P 4a 2a'
_space_group.transform_Pp_abc a,b,c;0,0,0

loop_
_space_group_symop_id
_space_group_symop_operation_xyz
1 x,y,z
2 x+1/2,-y,-z
3 -x,y+1/2,-z
4 -x+1/2,-y+1/2,z
5 -y,-x,-z
6 -y+1/2,x,z
7 y,-x+1/2,z
8 y+1/2,x+1/2,-z
9 -x,-y,-z
10 -x+1/2,y,z
11 x,-y+1/2,z
12 x+1/2,y+1/2,-z
13 y,x,z
14 y+1/2,-x,-z
```

```
15 -y,x+1/2,-z
16 -y+1/2,-x+1/2,z

loop_
_atom_type_symbol
Sn
O

loop_
_atom_site_label
_atom_site_type_symbol
_atom_site_symmetry_multiplicity
_atom_site_Wyckoff_symbol
_atom_site_fract_x
_atom_site_fract_y
_atom_site_fract_z
_atom_site_occupancy
_atom_site_fract_symmform
Sn1 Sn   2 c  0.2500000000  0.2500000000  0.5471700000  1.0000000000 0,0,Dz
Sn2 Sn   2 c  0.2500000000  0.2500000000  0.3504200000  1.0000000000 0,0,Dz
O1  O    4 f  0.7500000000  0.2500000000  0.3029600000  1.0000000000 0,0,Dz
O2  O    2 b  0.7500000000  0.2500000000  0.5000000000  1.0000000000 0,0,0
Sn3 Sn   2 c  0.2500000000  0.2500000000  0.7446200000  1.0000000000 0,0,Dz

# end of cif
```

S1.2.3 Bilayer

```
    # CIF file created by FINDSYM, version 7.1.3

data_findsym-output
_audit_creation_method FINDSYM

_cell_length_a     3.8215300000
_cell_length_b     3.8215300000
_cell_length_c     20.3659000000
_cell_angle_alpha  90.0000000000
_cell_angle_beta   90.0000000000
_cell_angle_gamma  90.0000000000
_cell_volume       297.4254679128

_symmetry_space_group_name_H-M "P 4/n 21/m 2/m (origin choice 2)"
_symmetry_Int_Tables_number 129
_space_group.reference_setting '129:-P 4a 2a'
_space_group.transform_Pp_abc a,b,c;0,0,0
```

```
loop_
_space_group_symop_id
_space_group_symop_operation_xyz
1 x,y,z
2 x+1/2,-y,-z
3 -x,y+1/2,-z
4 -x+1/2,-y+1/2,z
5 -y,-x,-z
6 -y+1/2,x,z
7 y,-x+1/2,z
8 y+1/2,x+1/2,-z
9 -x,-y,-z
10 -x+1/2,y,z
11 x,-y+1/2,z
12 x+1/2,y+1/2,-z
13 y,x,z
14 y+1/2,-x,-z
15 -y,x+1/2,-z
16 -y+1/2,-x+1/2,z

loop_
_atom_type_symbol
Sn
O

loop_
_atom_site_label
_atom_site_type_symbol
_atom_site_symmetry_multiplicity
_atom_site_Wyckoff_symbol
_atom_site_fract_x
_atom_site_fract_y
_atom_site_fract_z
_atom_site_occupancy
_atom_site_fract_symmform
Sn1 Sn   2 c  0.2500000000  0.2500000000  0.4386700000  1.0000000000 0,0,Dz
O1  O    4 f  0.7500000000  0.2500000000  0.3816900000  1.0000000000 0,0,Dz
Sn2 Sn   2 c  0.2500000000  0.2500000000  0.6754700000  1.0000000000 0,0,Dz

# end of cif
```

S1.2.4 Monolayer

```
# CIF file created by FINDSYM, version 7.1.3
```

```
data_findsym-output
_audit_creation_method FINDSYM

_cell_length_a     4.0793600000
_cell_length_b     3.5489100000
_cell_length_c     18.1506000000
_cell_angle_alpha  90.0000000000
_cell_angle_beta   90.0000000000
_cell_angle_gamma  90.0000000000
_cell_volume       262.7713455503

_symmetry_space_group_name_H-M "P 21/m 21/m 2/n (origin choice 2)"
_symmetry_Int_Tables_number 59
_space_group.reference_setting '059:-P 2ab 2a'
_space_group.transform_Pp_abc a,b,c;0,0,0

loop_
_space_group_symop_id
_space_group_symop_operation_xyz
1 x,y,z
2 x+1/2,-y,-z
3 -x,y+1/2,-z
4 -x+1/2,-y+1/2,z
5 -x,-y,-z
6 -x+1/2,y,z
7 x,-y+1/2,z
8 x+1/2,y+1/2,-z

loop_
_atom_type_symbol
Sn
O

loop_
_atom_site_label
_atom_site_type_symbol
_atom_site_symmetry_multiplicity
_atom_site_Wyckoff_symbol
_atom_site_fract_x
_atom_site_fract_y
_atom_site_fract_z
_atom_site_occupancy
_atom_site_fract_symmform
Sn1 Sn   2 b  0.2500000000  0.7500000000  0.5641450000  1.0000000000 0,0,Dz
```

```
O1  O    2 a  0.2500000000  0.2500000000  0.4922250000  1.0000000000 0,0,Dz

# end of cif
```

S1.3 Projected Density of States of SnO ML

In Fig. S2 we report the projected Density of States computed for SnO ML in the optimized orthorhombic cell (bottom panel) and in a tetragonal cell with $a = b$ fixed to the average of value of the othrhombic parameters. Energies are shifted so that deep states at very low energy align in the two structures.

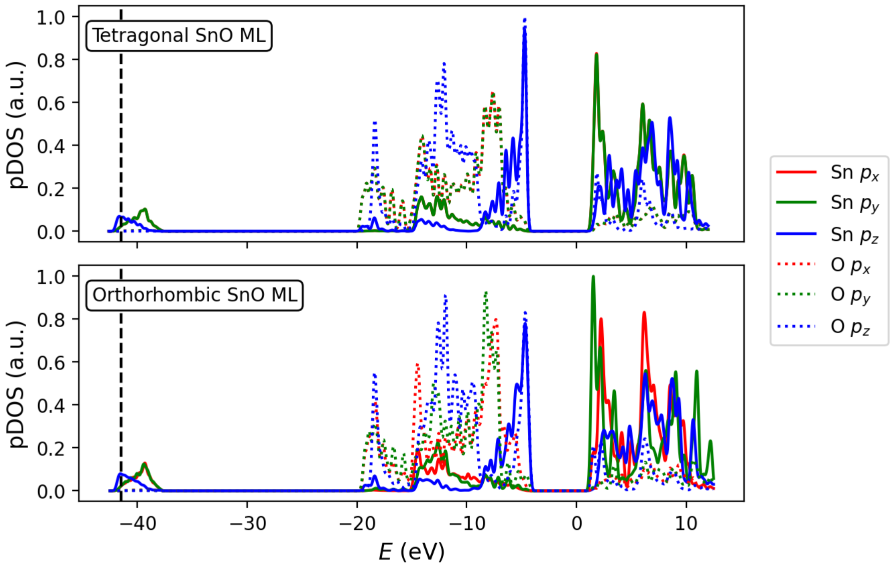

Figure S2: pDOS of SnO ML in the optimized orthorhombic geometry (bottom panel) and in a tetragonal geometry (upper panel).

S1.4 k-resolved projected density of states

The k-pDOS of states on Sn and O s, p_x, p_y, p_z orbitals are reported in Fig. S3 and Fig. S4 for SnO bulk and ML, respectively.

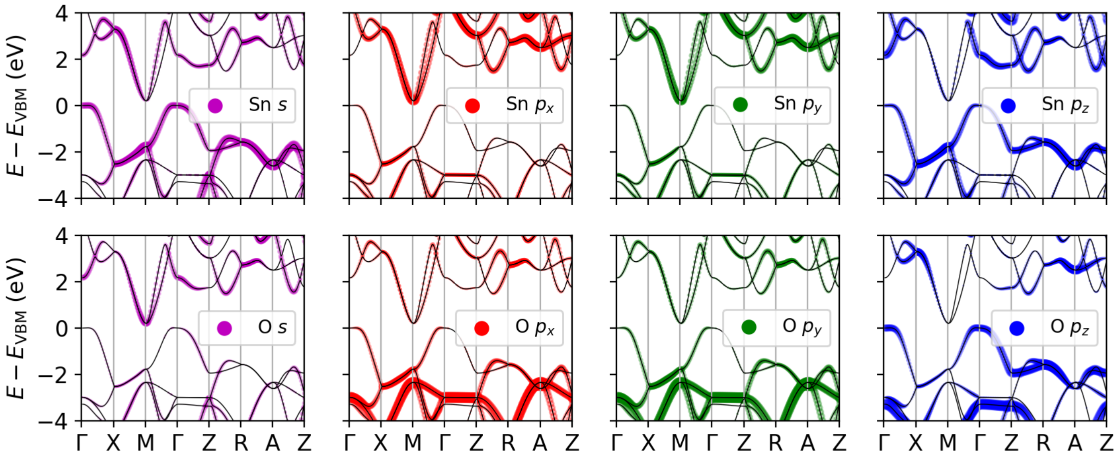

Figure S3: k-pDOS of SnO bulk.

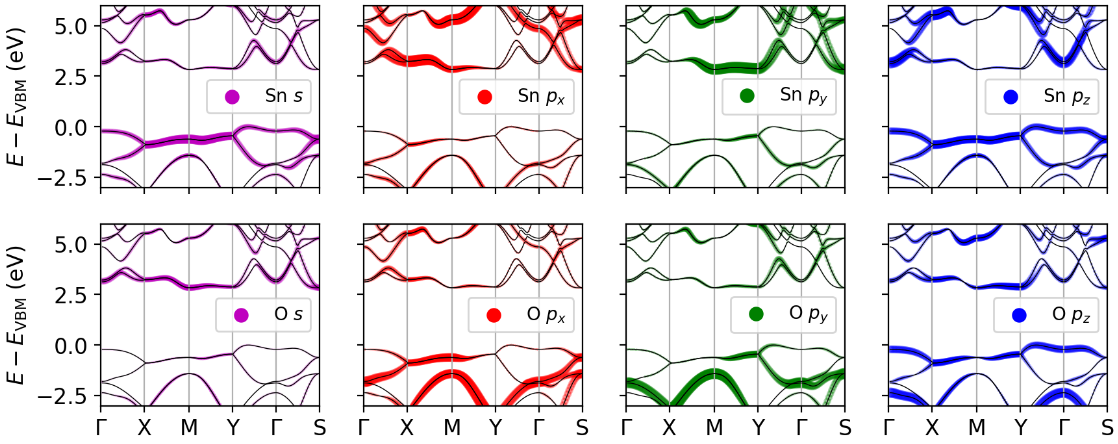

Figure S4: k-pDOS of SnO ML.

S1.5 Phonon dispersions

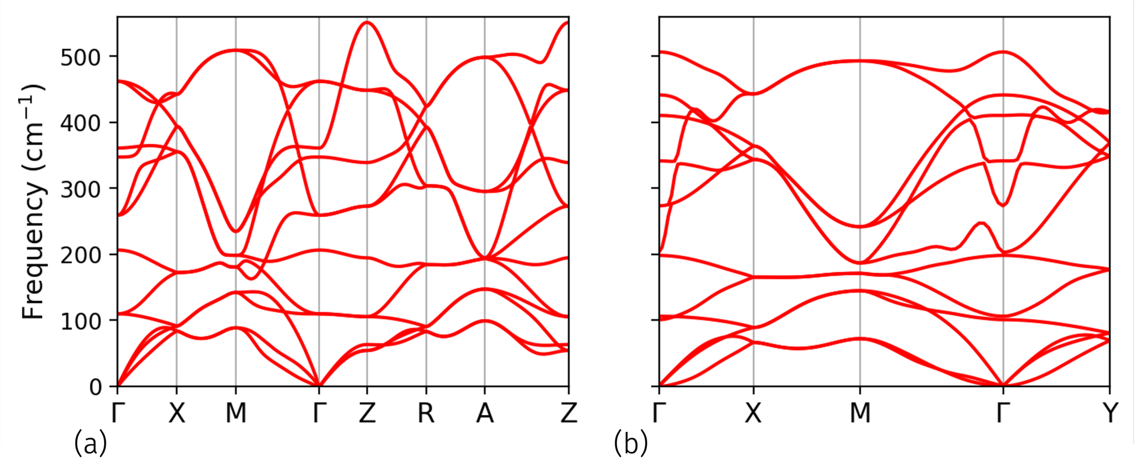

Figure S5: Phonon dispersions of SnO bulk (a) and ML (b).

S2 Convergence of MBPT calculations

S2.1 k-point mesh in G_0W_0

The convergence of G_0W_0 calculations with respect to the k-point mesh was established by studying the variation of the direct bandgap at the Γ point of both ML and bulk while keeping all other parameters (number of bands in the screening N_b, energy cutoff on the dielectric matrix size $E_{\mathbf{G}}$ and number of bands in Σ^c, N_b^c) fixed. For the bulk system: $N_b = 500$, $E_{\mathbf{G}} = 5$ Ry, $N_b^c = 200$. For the ML system: $N_b = 1000$, $E_{\mathbf{G}} = 5$ Ry, $N_b^c = 200$.

The bandgap evolution with the number of k-points N_k for both systems is reported in Fig. S6

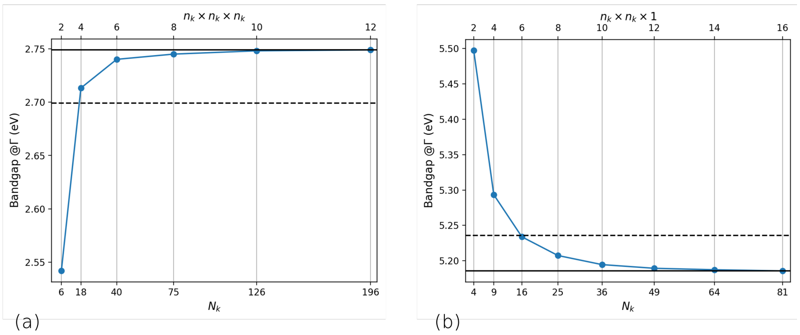

Figure S6: Convergence of the bulk (a) and monolayer (b) bandgap at Γ with number of k-points N_k.

S2.2 Dielectric screening in G_0W_0

Given that convergence behavior with respect to N_b and $E_{\mathbf{G}}$ is generally independent of k-point grid density, we investigated the convergence of these two parameters while fixing the k-point grid density to $4 \times 4 \times 4$ for bulk and $6 \times 6 \times 1$ for ML.

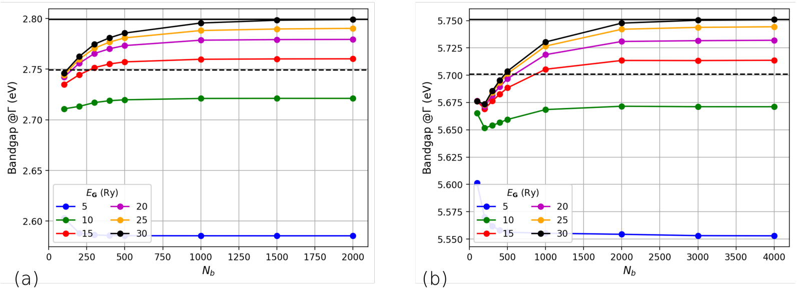

Figure S7: Bandgap value at Γ with respect to N_b and $E_{\mathbf{G}}$ for bulk (a) and ML (b).

The evolution of the bandgap with N_b and $E_{\mathbf{G}}$ for SnO bulk and ML is reported in Fig. S7 (a) and (b), respectively.

S2.3 Convergence of BSE calculations

The convergence analysis of Bethe-Salpeter Equation (BSE) calculations with respect to the k-point grid ($n_k \times n_k \times n_k$ for bulk and $n_k \times n_k \times 1$ for ML), energy cutoff $E_{\mathbf{G}}$ and the number of bands in static screening N_b, as well as the number of valence (n_v) and conduction (n_c) bands in the BSE Hamiltonian for SnO is presented in Fig. S8. The results for the bulk system are shown in the upper panels, while those for the ML system are displayed in the lower panels.

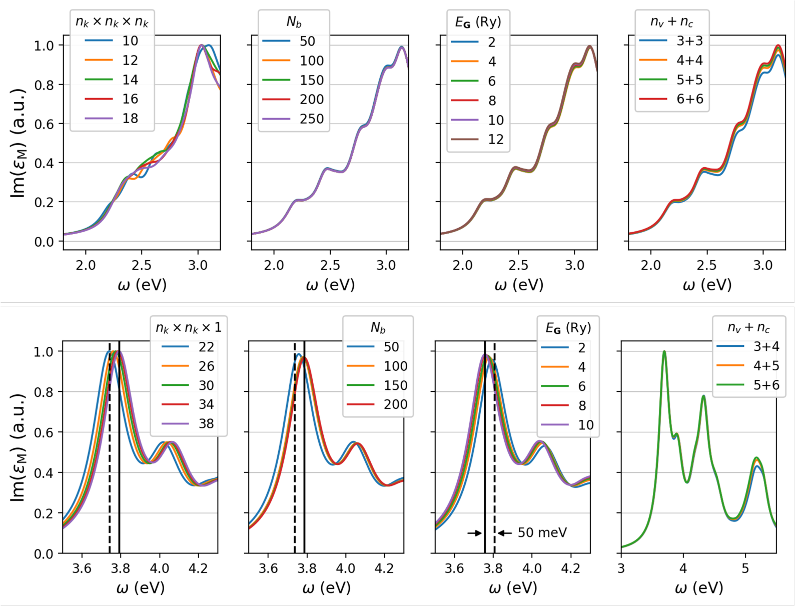

Figure S8: Convergence of BSE spectra for SnO bulk (upper panels) and ML (lower panels).

Following this analysis, for the bulk system we chose an $18 \times 18 \times 18$ k-point mesh, $N_b = 50$, $E_{\mathbf{G}} = 50$ Ry and $n_v = n_c = 4$. For SnO ML we employed a $35 \times 35 \times 1$ grid, $N_b = 100$, $E_{\mathbf{G}} = 10$ Ry and $n_v = 4$, $n_c = 5$.

References

[1] Harold T. Stokes and Dorian M. Hatch. *FINDSYM*: program for identifying the space-group symmetry of a crystal. Journal of Applied Crystallography, 38(1):237–238, Feb 2005.

[2] Harold T. Stokes and Dorian M. Hatch. Findsym, isotropy software suite. `https://stokes.byu.edu/iso/findsym.php`. Accessed: 2025-01-07.
